# Food allergies around the world

**DOI:** 10.3389/fnut.2024.1373110

**Published:** 2024-06-13

**Authors:** Gary Wing-Kin Wong

**Affiliations:** Department of Paediatrics, Faculty of Medicine, The Chinese University of Hong Kong, Hong Kong SAR, China

**Keywords:** food allergy, risk factor, anaphylaxis, epidemiology, global

## Abstract

The increase in the prevalence of food allergy has been considered as the second wave in the allergy epidemic following the first wave of increase in asthma and allergic rhinitis. It is well known that the prevalence of allergic conditions would follow economic development and urbanization in many countries or regions. In developed countries, one in three children suffered from at least one allergic disorder and these conditions include food allergy, eczema, allergic rhinitis and asthma. Food allergy is very often the first allergic manifestation affecting infants and young children. The exact etiologies are not known. The clinical manifestations ranged from a simple rash or an itch around the mouth, to the more severe manifestations of angioedema and potentially fatal anaphylaxis. Among all cases of childhood anaphylaxis, food is the most common cause. The common allergens resulting in food allergies in developed countries include egg, milk, fish, wheat, peanuts and tree nuts. However, there are marked variations in the patterns of food allergens in developing countries. In line with the epidemiology of asthma, food allergy is also much less common in rural areas. Clear understanding of reasons explaining the disparity of food allergies between urban and rural population would pave the way to the development of effective primary prevention for food allergy.

## Introduction

The first wave of the allergy epidemic began more than 50 years ago with the increase in the prevalence of asthma and allergic rhinitis. Food allergy has been considered as the second wave of the allergy epidemic started about 20 years ago. Similar to the epidemiology of asthma, countries with high prevalence rates of asthma were also found to have higher prevalence of food allergy (1, 2). Although there have been many epidemiology studies of food allergies around the world, accurate determination of the true prevalence of food allergies is very difficult. Most published studies have only used questionnaires to ascertain possible allergic symptoms related to food allergies, and only a small proportion of studies included objective testing such as skin prick test, serum specific IgE measurements or food challenge (3). The lack of objective testing and use of different methodologies make comparison of the data from different published epidemiology studies very difficult. Nevertheless, the patterns of food allergies appeared to be different across the world (2). Early guidelines from many countries recommended dietary avoidance or delayed introduction of allergenic foods especially in high risk infants. However, there have been several prospective randomized trials showing that early introduction of allergenic foods may be beneficial in preventing food allergies (4, 5). Subsequently, guidelines from around the world now recommend early introduction rather than avoidance of allergenic foods in infancy (6, 7). The continuing changes in the environment including dietary changes will shape the epidemiology of food allergies around the world. Clear understanding of how these environmental factors interact with the immune system resulting in the manifestations of food allergies will lead to development of effective primary preventive strategies.

## Epidemiology of food allergy

Food allergy is defined as an adverse health effect due to a specific immune response that reproducibly occurs upon exposure to a specific food. Such adverse effects can be sub-divided in to two major types, IgE-mediated or non-IgE-mediated. Over the past few decades, there have been many epidemiology studies of food allergy around the world, and most of these studies evaluated the IgE-mediated type of food allergy using non-standardized methodologies (3, 8). Due to the complexity of evaluation of serum IgE in large-scale population studies, most published studies have only used questionnaires to evaluate the symptoms of food allergy without objective testing. However, many other diseases may have symptoms mimicking those of true food allergies. Studies using only questionnaires frequently overestimated the true prevalence of food allergy. Furthermore, the lack of standardization of various studies made comparison of the results difficult, if not impossible. Nevertheless, the prevalence rates and the patterns of food allergens appeared to be widely different across the world (2, 9, 10).

The most common food allergens include cow’s milk, wheat, egg, peanuts, shellfish, fish, soy, and tree nuts. In some parts of the world such as some Asian countries, other exotic allergens such as insects and bird’s nest can also induce food allergies (2). A telephone survey conducted across 10 European countries revealed a wide variation in the prevalence of food allergy among children in these countries. In this study, parents or guardians were interviewed to ascertain possible symptoms of food allergies of their children (11). Finland was found to have the highest reported prevalence of 11.7% while Austria was lowest at 1.7%. Cow’s milk, fruits, and egg were the top three most common offending foods. The European Community Respiratory Health Survey also revealed a wide variation of symptoms of adverse food reactions in European adults. The highest rate was reported in Melbourne, Australia, while it was lowest at 4.6% in Spain (12). A meta-analysis of 51 studies across the world revealed marked variations of the prevalence of food allergies ranging from 3 to 35% (3). However, the average prevalence rate of food allergy was only 3% if only studies with objective testing were included. These findings suggest marked over-estimation of the true prevalence by studies using questionnaires only. Food allergy has been reported to be common in the United States, affecting up to 8% of children and 10% adults. These studies were based on questionnaires only and might potentially overestimated the true prevalence (13, 14). The HealthNuts study from Melbourne, Australia used food challenge to document true food allergies. These Australian infants had a very high prevalence of food allergies (3% to peanuts, 8.9% to raw egg) (15).

In the past two decades, exposure to rural environments has been persistently documented to be a major protective factors against the development of allergies (16). Food allergy is no exception. A comparative study using both questionnaire and skin prick test were conducted in South Africa to evaluate the prevalence of allergic diseases in both urban and rural children (17). A total of 1,185 urban and 398 rural toddlers aged 12–36 months were recruited for this study. The prevalence of food allergy was 2.3% in the urban population while it was only 0.5% in the rural population. The recent EuorPrevall-INCO study evaluate a large sample of 35,549 school children from China, Russia, and India using standardized questionnaires along with skin prick test and serum specific IgE measurement demonstrated that children from the highly urbanized city of Hong Kong had a prevalence of food allergy 3 times higher than those of children from mainland China (10). Furthermore, among children recruited from Hong Kong, those born and raised in Hong Kong had a prevalence of food allergy three times higher than those children born in mainland China and subsequently migrated to Hong Kong. Given the same genetic background of these two groups of children, early life exposures most likely have affected the early development of the immune system leading to differences in the subsequent manifestations of food allergy. A recent comparative study also demonstrated that Asians born in Australia had markedly higher prevalence of food allergy when compared to Asians from Singapore (18, 19). Many factors may potentially be important in explaining the disparity, and the potential factors are summarized in Table 1. Further studies are needed to evaluate how various environmental exposures and dietary factors might influence the early maturation of the immune system resulting in protection against the development of food allergy.

**Table 1:** Potential factors associated with the development of food allergy.

Factors	Protection or risk factor for food allergy
Urban vs. rural	Rural environment may be associated with protection
Breast feeding	Protection: exclusive breast feeding for at least 6 months
Eczema	Risk: poorly control eczema associated with food sensitization through the inflamed skin
Introduction of solids	Protection: introduction of complementary foods at around 6 Months
Maternal diet	No evidence for food avoidance during pregnancy
Formula	Inconsistent evidence for the use of hydrolyzed formula for the prevention food allergy
Probiotics	Inconsistent results for the prevention of food allergy
Vitamin D	Inconsistent and requires further studies

## Spectrum of food allergic reactions

The manifestations of food allergy vary widely from a very mild itch or redness on the skin, urticaria affecting a large area on the body, sneezing, itchy and watery eyes to the more severe symptoms such as difficulty with breathing and hypotension (19). Pollen-food allergy syndrome is a specific type of food allergy when the primary sensitizer is pollen such as birch pollen (20).

Due to similarity to other allergens found in fruits and vegetables, binding of the food allergens with the cross-reacting IgE would result in various symptoms of food allergy. The mildest form of pollen-food allergy syndrome typically associated with symptoms including itchy mouth, swelling of the lips and tongue. Such mild reaction is termed oral allergy syndrome, but more severe reactions including anaphylaxis can occur in patients with pollen-food allergy syndrome. Anaphylaxis is the most severe manifestation of food allergy, and appropriate treatment must be provided promptly to prevent mortality. Hospital admission data from across the world in the past 2 decades revealed significant increase in the number of food induced anaphylaxis admitted to hospitals in the United Kingdom, USA, Australia and Hong Kong (21–24). The increase was found to be highest in Australian children under 4 years of age. From 1998 to 2012, the Australian hospital admission rate due to food induced anaphylaxis in children under 4 years has increased from 7.3 to 21.7 per 100,000 per year (21). Hospital admission data from Hong Kong also showed a doubling of anaphylaxis admission in 2019 when compared with data in 2009 (24). In order to optimize the clinical care of patients with food allergy, appropriate counseling of patients at risk for development of severe food allergic reactions is extremely important. However, there are no reliable features that can accurately predict the severity of food allergy. Many studies have showed that prior anaphylaxis was not a good predictor of the future risk. In fact, fatality associated with food induced anaphylaxis often occurred in patients with only prior mild reactions. Many co-factors are known to be associated with more severe reactions, such as concomitant poorly controlled asthma, medications (ACE inhibitor, beta-blockers), febrile illness, and menstruation (25, 26). Exercise is a well-known trigger typically associated with wheat allergy due to IgE sensitization to omega-5-gliadin (27). These patients usually can tolerate a small dose of wheat, but exercise would dramatically reduce the threshold and result in severe reactions. Due to the limitation of accurate prediction of severe reactions, all patients with food allergy should be educated to recognize and manage severe food allergic reactions. Although many different foods can result in food allergic reactions, they tend to differ in different regions of the world. In Europe, North America, and Australia, peanut, tree nuts, and cow’s milk are the most common foods resulting in food induced anaphylaxis while shellfish and seafood are more common in Asia and Latin America (2, 25). The exact reasons explaining such differences in the pattern of food allergens are not clear. In Hong Kong, shrimp has been found to be the most common food resulting in anaphylaxis seen in the emergency department (28). However, variations of environmental factors and dietary exposures are likely important in causing some of the differences. In rural China, cockroach has been found to be a major cross-reactive allergen source in shrimp-sensitized children (29). Further studies are needed to clearly understand how environmental exposures may influence the manifestations of food allergy in different parts of the world.

## Recent development and updates of food allergy guidelines

Allergen avoidance, whether during pregnancy or in early infancy, has been thought to be useful in reducing the risk of sensitization, and hence the risk of allergy. In the past, many guidelines from around the world recommended a variety of avoidance measures during pregnancy or in early infancy especially for high risk infants (30–32). For example, it was recommended that infants should avoid egg and milk for the first year of life, and peanuts, tree nuts, shellfish till 2–4 years of age. However, recent randomized controlled trials have documented that avoidance of food allergens during pregnancy could not reduce the risk of food allergy in their offspring. Furthermore, there have been several prospective randomized clinical trials testing whether food avoidance in early infancy was effective in preventing subsequent food allergies (4, 5). The Learning Early About Peanut (LEAP) trial recruited 640 high risk infants and randomized them to consume 6 g of peanut protein weekly or to avoid peanuts for the first 5 years of life (4). At 5 years of age, peanut allergy documented by food challenge in the avoidance group was 17.2% as compared with the 3.2% in the intervention group. In the Enquiry About Tolerance (EAT) study, more than 1,300 infants were recruited and randomized to introduction of six allergenic foods (peanut, egg, cow’s milk, sesame, fish, and wheat) starting at 6 month of age (5). The children were followed up till 3 years of age for assessment of possible food allergy to the six allergenic foods. In the per-protocol analysis, the primary outcome was significantly lower in the early introduction group (2.4%) than in the standard-introduction group (7.3%), but the difference was not significantly different in the intention-to-treat analysis. Adherence to this challenging protocol was very low at 42.8% as many parents found it very difficult to feed so many different types of solid foods to their young infants. These clinical trials clearly demonstrated that early consumption rather than delayed introduction was likely more beneficial as primary preventive strategy. Since the publication of these clinical trials, guidelines from around the world have been updated to recommend early introduction of allergenic food at around 6 months of age, and maternal dietary restrictions during pregnancy are no longer recommended (6, 7).

The remaining question is how to translate these trial findings into the real world. A recent study from Australia has been conducted comparing the prevalence of peanut allergy in one-year-old infants before and 2 years after the guideline recommendation was changed (33). A total of 7,209 infants were recruited with 1933 in 2018–2019 and 5,276 in 2007–2011. Despite earlier introduction of peanut in the later cohort, there was no significant change in the prevalence of peanut allergy across this population. Further studies are needed to determine how to introduce allergenic foods early in infants in the community level, and evaluate other potential environmental factors which may affect the manifestation of food allergy. One should also note that the prevalence of peanut allergy is extremely rare in many Asian countries such as Thailand and China (10). A birth cohort from Singapore documented a very low prevalence of food allergy despite rather late introduction of allergenic foods (34). Therefore, a consensus statement from the Asia Pacific Academy of Pediatric Allergy, Respirology & Immunology recommends that it is not necessary to delay introduction of solids, but more studies are needed to determine the role of early introduction of various allergenic foods in populations with a very low background prevalence of food allergy (35).

## Discussion

There is no doubt that food allergy is becoming a major problem especially in the developed countries such as United Kingdom, United States, and Australia in the past two decades. The exact reasons driving the increase in the prevalence are not clear. Evidence is building up that early consumption as opposed to delayed introduction may be a more effective approach to prevent the subsequent development of food allergy. On the other hand, there are potentially many lessons we can learn from countries where the background prevalence of food allergy is very low, and yet early introduction of allergenic foods in these countries is not the norm. Similar to the manifestations of other atopic conditions such as asthma or allergic rhinitis, the development of food allergy is the result of the complex interactions between environmental exposures and genetic influences. The disparity of food allergy in populations with the same genetic background brought up in different environments clearly illustrates the importance of environmental exposures which include dietary variations in shaping the development of food allergy. Clear understanding of the underlying reasons explaining the variations of food allergy epidemiology around the world will provide clues for developing effective primary preventive strategies.

## Author contributions

GW-KW: Conceptualization, Formal analysis, Funding acquisition, Methodology, Writing – original draft, Writing – review & editing.
